# Compliance With Clozapine Monitoring Standards and Plasma Assay Utility in a Community Mental Health Team

**DOI:** 10.1192/bjo.2026.11732

**Published:** 2026-06-30

**Authors:** Tlhompho Gwen Ditedu, Inderpreet Jassal

**Affiliations:** Sheffield Health Partnership University NHS Trust, Sheffield, United Kingdom

## Abstract

**Aims:**

To evaluate adherence to the Sheffield Health Partnership University NHS Trust (SHPU) clozapine monitoring Standard Operating Procedure (SOP) which requires that patients on clozapine have their plasma levels checked yearly at their annual clozapine review.

To investigate the clinical utility of annual plasma assays in informing dose adjustments.

**Methods:**

A retrospective audit was conducted on a random sample of 30 patients drawn from a total population of 108 clozapine-treated patients at the South Recovery Community Mental Health Team (CMHT). To be included, patients had to be under the South Recovery CMHT and on clozapine for at least one year at point of data collection. Data were extracted from an electronic record system, focusing on the completion of annual patient reviews, the requesting of clozapine assays as per protocol, and subsequent clinical actions taken based on results of these assays.

**Results:**

80% of patients were reviewed by a psychiatrist within a 12-month period. Full compliance with the SOP was achieved in 40% of the sample.

Major barriers to compliance included:

- **Psychiatric Reviews:** 20% were overdue by at least four months
- **Assay Requests:** 23% were not marked as required, often without documented reasoning.
- **Completion:** 17% of requested assays were never performed, frequently due to coordination issues between the CMHT and General Practice (GP) services.

Regarding the clinical utility of clozapine assays: Of the nine results outside the therapeutic threshold levels (six low, three high), only three led to a change in dose. This illustrated that the decision to change a patient’s clozapine dose does not entirely depend on the assay result. There can be other factors that determine whether a dose is changed or not such as patient response and preference.

**Conclusion:**

While annual plasma assays led to dose changes in only a small minority of patients, they remain a critical safety tool for identifying extreme outliers and monitoring adherence. Current compliance is hindered by administrative issues. Adherence could be improved by automating review reminders and synchronizing plasma assays with annual physical health blood draws to reduce the risk of them being missed. Clearer protocols are required to define responsibility for requesting and chasing blood results as currently this remains ambiguous. Further investigation with a larger sample size is needed to fully quantify the impact of assays on psychiatrist decision-making.

